# Function of the N-terminal segment of the RecA-dependent nuclease Ref

**DOI:** 10.1093/nar/gku1330

**Published:** 2015-01-23

**Authors:** Angela J. Gruber, Tayla M. Olsen, Rachel H. Dvorak, Michael M. Cox

**Affiliations:** Department of Biochemistry, University of Wisconsin-Madison, Madison, WI 53706, USA

## Abstract

The bacteriophage P1 Ref (recombination enhancement function) protein is a RecA-dependent, HNH endonuclease. It can be directed to create targeted double-strand breaks within a displacement loop formed by RecA. The 76 amino acid N-terminal region of Ref is positively charged (25/76 amino acid residues) and inherently unstructured in solution. Our investigation of N-terminal truncation variants shows this region is required for DNA binding, contains a Cys involved in incidental dimerization and is necessary for efficient Ref-mediated DNA cleavage. Specifically, Ref N-terminal truncation variants lacking between 21 and 47 amino acids are more effective RecA-mediated targeting nucleases. We propose a more refined set of options for the Ref-mediated cleavage mechanism, featuring the N-terminal region as an anchor for at least one of the DNA strand cleavage events.

## INTRODUCTION

Encoded by bacteriophage P1, the recombination enhancement function (Ref) protein is a 21-kDa RecA-dependent HNH endonuclease that can be targeted to produce double-strand breaks (DSBs) at any desired DNA sequence. Early reports showed that expression of Ref enhanced recombination events in a RecA- and RecBCD-dependent manner in *Escherichia coli* (1–3). Deletion of the *ref* gene in bacteriophage P1 has no measurable effect on lysogeny or lytic cycles, and an *in vivo* role for *ref* has not yet been elucidated (3).

Unlike many other temperate phages, the P1 prophage is maintained as a low copy number, autonomous plasmid. The linear double-stranded (linear ds) genome is cyclized upon infection. Typically, this process occurs via the phage-encoded Cre-lox site-specific recombination system, but it can also occur by RecA-mediated homologous recombination (4). Ref may play a role in this RecA-dependent cyclization of the genome (5). Some phage HNH proteins partner with terminases in the genome packaging reaction (6). However, Ref exhibits no homology to the terminase-associated HNH protein nucleases.

An effort to characterize the bacteriophage P1 Ref protein as a suspected RecA-regulator protein revealed that Ref is actually a RecA-dependent, HNH-family endonuclease (7). Ref has the novel property of only cleaving DNA to which RecA protein is bound. In the absence of any cofactors or proteins, Ref will bind to both single-stranded DNA (ssDNA) and double-stranded DNA (dsDNA), but no DNA cleavage occurs. In the presence of RecA, ATP and Mg^2+^, Ref produces extensive degradation of ssDNA (7). The ATP and Mg^2+^ are required for an active RecA nucleoprotein filament, and this in turn is necessary for Ref nuclease activity. More importantly, Ref will create DSBs in a small target area within a RecA formed displacement loop (D-loop). RecA forms a nucleoprotein filament on an oligonucleotide (100–150 nucleotides (nt) in length) and initiates strand invasion in the homologous region of a dsDNA. In strand invasion, the RecA-bound oligonucleotide base pairs to the complementary strand, and the other strand of the duplex is displaced. Ref then cleaves the paired and displaced strands of the targeted duplex DNA sequentially within the D-loop region (8). The initial cleavage of the paired strand is relatively fast, does not require ATP hydrolysis by RecA and is promoted by the Ref active site mutant H153A (8). The second cut on the displaced strand is produced at a much slower rate, requires RecA-mediated ATP hydrolysis and is not promoted by Ref H153A (8). The two cleavage events thus appear to be mechanistically distinct.

The Ref protein (21 kDa; 186 amino acids) consists of a 76 residue amino-terminal domain and a 110 residue C-terminal globular domain. The structure of the C-terminal globular domain of Ref has been determined to 1.4-Å resolution (PDB ID: 3PLW) (7). The asymmetric unit of Ref contains a monomer and two stably bound Zn^2+^ ions. The structure features an HNH domain defined by a ßßα metal-binding core. Outside of this core element, HNH nucleases are structurally and catalytically diverse, including group I and II homing endonucleases, transposases, restriction endonucleases and bacterial colicins (9). Colicins digest dsDNA non-specifically, while homing endonucleases create nicks or double-stranded breaks at specific DNA sequences. Ref does not exhibit any dominant sequence specificity, however, there is a preference for a phosphodiester bond to the 5′ side of a pyrimidine base (8).

In contrast, there is limited characterization of the N-terminal domain. Electron density for the N-terminal region was absent in the crystal structure (7), suggesting disorder. In the past decade, attention has been placed on intrinsically disordered proteins or parts of proteins (10–12). In many cases, protein domains that are intrinsically disordered in solution become ordered upon binding to a ligand. The sequence of Ref N-terminal domain exhibits recognized hallmarks of an intrinsically disordered region (10–13), particularly in its high concentration of amino acid side chains that should be charged at neutral pH, with 25 positively charged and 9 negatively charged among the 76.

Here, we characterize key aspects of structure-function in the Ref protein, with a focus on the active oligomeric form and the function of the disordered N-terminal 76 amino acids. Although a complete N-terminal domain deletion still retains nuclease activity, a 100-fold higher concentration is required to reach wild-type (WT) cleavage levels (7). This early work also found that when the N-terminal domain was absent, the Ref protein no longer bound to DNA. Visual inspection of the sequence revealed a possible signature charge distribution motif (imperfectly repeated), which was used to guide the design of N-terminal deletion variants. We demonstrate that partial removal of the N-terminal region leads to an enhancement of targeted Ref cleavage at D-loops. An understanding of N-terminal domain function is critical to further development of the Ref system in biotechnology applications, and that is the primary focus of the current study.

## MATERIALS AND METHODS

### DNA substrates

The M13mp18 circular ssDNA (7249 nt) was prepared as described previously (14,15). The M13mp18 circular dsDNA was prepared as described previously (14–16). All DNA concentrations are given in micromolar nt (μM nt). Oligonucleotides were purchased from Integrated DNA technologies. Sequences of oligonucleotides used in this study can be found in Supplementary Materials and Methods.

### Cloning ref and ref variants

The WT *ref* gene from bacteriophage ϕW39 was not available, as stocks of this phage no longer exist. The ϕW39 *ref* gene was reconstructed to match the reported sequence by mutagenesis of the P1 *ref* gene in pEAW584 (WT P1 *ref* in protein expression vector pET21A (Novagen)). Detailed cloning procedures for all Ref variants can be found in the Supplementary Materials and Methods.

### Proteins

The *E. coli* RecA E38K protein was purified as described previously (Ronayne, 2014). The P1 WT Ref and P1 ΔN76 proteins were purified as described previously (7). All Ref variants were purified in their native form using standard chromatography procedures, and quantified, as described in the Supplementary Materials and Methods. The mass of all variants was confirmed by mass spectrometry. All proteins were stringently tested for exonuclease and endonuclease contamination and all were free from detectable nuclease activity in the absence of RecA protein.

### Fluorescence polarization DNA binding assay

Ref proteins at 0.5–10 000 nM were incubated with 2 nM AJM25 71mer ssDNA or AJM25 annealed with AJG52 71mer to make a linear 71 bp dsDNA substrate in 25 mM Tris-acetate (pH 8.5), 3 mM potassium glutamate, 15 mM magnesium acetate and 5% w/v glycerol at room temperature for 15 min. Fluorescence anisotropy (FA) was measured at 25°C, using a Tecan Infinite M1000 instrument with 470-nm excitation and 535-nm emission wavelengths for at least three replicates. The average FA values were plotted with one standard deviation of the mean shown as error. Prism software was used to convert FA values to the percent of DNA bound and apparent dissociation constants were determined using one-site, specific binding with Hill coefficient.

### Circular ssDNA nuclease assay

Ref proteins were tested for nuclease activity as previously described (7). See details in Supplementary Materials and Methods.

### Nuclease site-specific targeting assay

Ref proteins were tested for targeted nuclease activity in assays as previously described (8). See details in Supplementary Materials and Methods.

### Non-reducing sodium dodecyl sulphate-polyacrylamide gel electrophoresis (SDS-PAGE)

All Ref proteins were dialyzed into 20 mM sodium phosphate (pH 7.5), 200 mM sodium choloride and 10% glycerol to remove DTT present from purification. Each reaction contained 6.5 μg Ref protein, 50 mM sodium phosphate (pH 7.5), 75 mM sodium chloride and in indicated reactions 10 mM dithio (DTT). Non-reducing cracking buffer (80 mM Tris-HCl pH 6.8, 2% SDS, 10% glycerol, 0.2% bromophenol blue) was added to each sample and then run on a 4–15% gradient SDS-PAGE.

## RESULTS

### A repeating pattern in the charge distribution in the N-terminal domain

An examination of sequence databases revealed few homologs of the P1 Ref protein, essentially all encoded by bacteriophages or cryptic bacteriophage remnants closely related to P1. We investigated P1 Ref homologs from bacteriophages P7 and ϕW39 (17–19) to determine if targeting efficiency could be increased relative to reactions previously characterized with the Ref protein from bacteriophage P1 (7). The ϕW39 Ref protein proved to be tractable, identical to P1 Ref in its RecA-dependence, and at least as active as P1 Ref in all RecA-dependent nuclease assays. The P1 and ϕW39 Ref enzymes are 95% identical in amino acid sequence. One difference at residue 11, ultimately helped define oligomeric properties. The two proteins are used interchangeably in this study, and the N-terminal deletion variants were constructed in ϕW39 Ref.

The P7 Ref protein has 13 amino acid changes in comparison to P1 WT Ref as well as an additional 30 amino acids on the C-terminus. It was insoluble in several conditions tested (unpublished data). As we were unable to locate viable stocks of bacteriophage P7 (the P7 *ref* gene was reconstructed based on the published sequence), this protein was not pursued.

**Figure 1.**
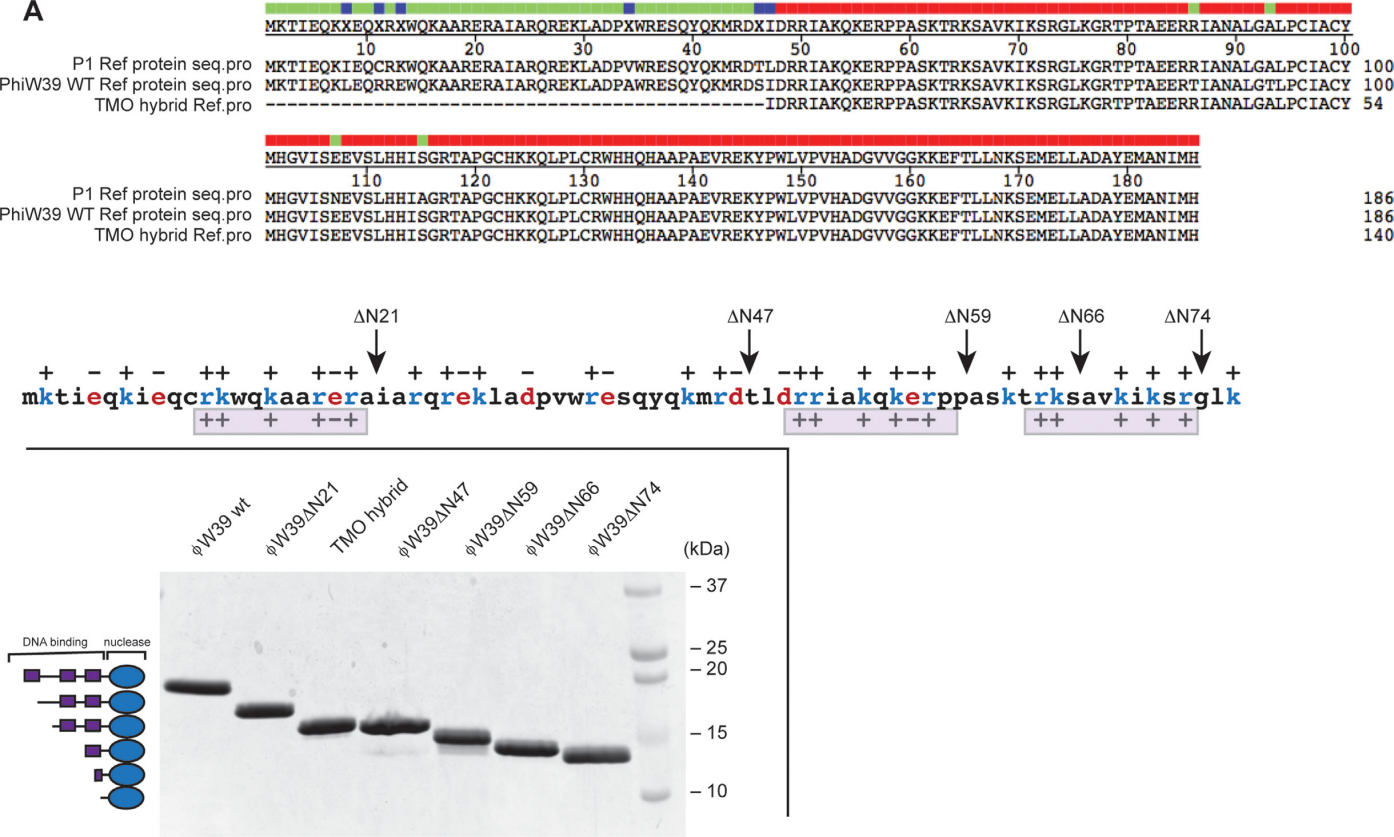
Sequence alignment and overview of Ref truncation variants based on charge distribution. (A) Protein sequence alignment of P1 WT Ref, ϕW39 WT Ref and TMO hybrid Ref proteins. There are 10 amino acid differences between P1 Ref and ϕW39 Ref. TMO hybrid is a truncated version with a mix of P1 Ref and ϕW39 Ref amino acids at heterologous positions. (B) Schematic of protein sequence and charge distribution of unstructured N-terminal 76 amino acids of P1 WT Ref. C-terminal core nuclease domain crystal structure is also included (PDB: 3PLW (7)). Based on the signature charge distribution (++ + +-+) pattern shown below the sequence, various truncations of ϕW39 Ref were cloned and purified. (C) Schematic of Ref truncations and image of a Coomassie-stained SDS-PAGE gel containing ∼4 ug of each construct, left and right panels, respectively. All proteins were soluble and purified in native form.

The ϕW39 WT Ref has 10 amino acid changes in comparison to the P1 Ref (6 of these in the unstructured N-terminus), but no additional C-terminal amino acid residues (Figure 1A). Stocks of ϕW39 bacteriophage were also unavailable. Therefore, the reported sequence of ϕW39 Ref was reconstructed by directed mutagenesis of the P1 *ref* gene. An intermediate in the construction of the ϕW39 Ref, referred to as the TMO hybrid Ref protein, is also included in this study and was serendipitously cloned and purified (Figure 1A). Of the four amino acid residues in the C-terminal core domain that differ between the two proteins, the TMO hybrid has only two of the changes characteristic of the conversion of P1 Ref to ϕW39 Ref, N107E and A115S (Figure 1A). The TMO hybrid protein also lacks 47 amino acid residues from the N-terminus, due to a precise but apparently spontaneous protein degradation event that occurred during purification. Protease inhibitors were used in subsequent purification trials to prevent any further spontaneous degradation, and molecular weights of all purified proteins were verified by mass spectrometry.

Examining the N-terminus region more closely, we identified a potential signature charge pattern (consensus: ++ + +-+) repeated imperfectly three times as shown in Figure 1B and Supplementary Figure S1. This pattern guided the design of a series of specific truncation variants with one or more of these putative motifs removed (Figure 1B). The Ref protein truncations purified included ϕW39 ΔN21, ϕW39 ΔN47, ϕW39 ΔN59, ϕW39 ΔN66 and ϕW39 ΔN74 (Figure 1C). The P1 ΔN76 Ref that was previously purified (7) was included in many assays for purpose of comparison. We attempted, unsuccessfully, to purify a variant including only the N-terminal 76 amino acid residues of P1 Ref (Ref ΔC110). The highly charged nature of the N-terminal region renders the protein largely insoluble.

### Removing charge motifs in the N-terminal domain decreases DNA-binding affinity

The P1 ΔN76 Ref no longer exhibits DNA-binding activity (7), indicating the N-terminus is essential for DNA binding. We employed fluorescence polarization with labeled DNA to determine apparent dissociation constants (*K*_d,app_), thereby measuring the DNA binding function of this domain. One key parameter needed to determine true *K*_d_ values, the DNA binding site size of Ref, is currently unknown and may change with each truncation variant. Our data analysis was thus limited to reporting apparent (*K*_d app_) values that provide a measure of relative DNA binding affinity.

**Figure 2.**
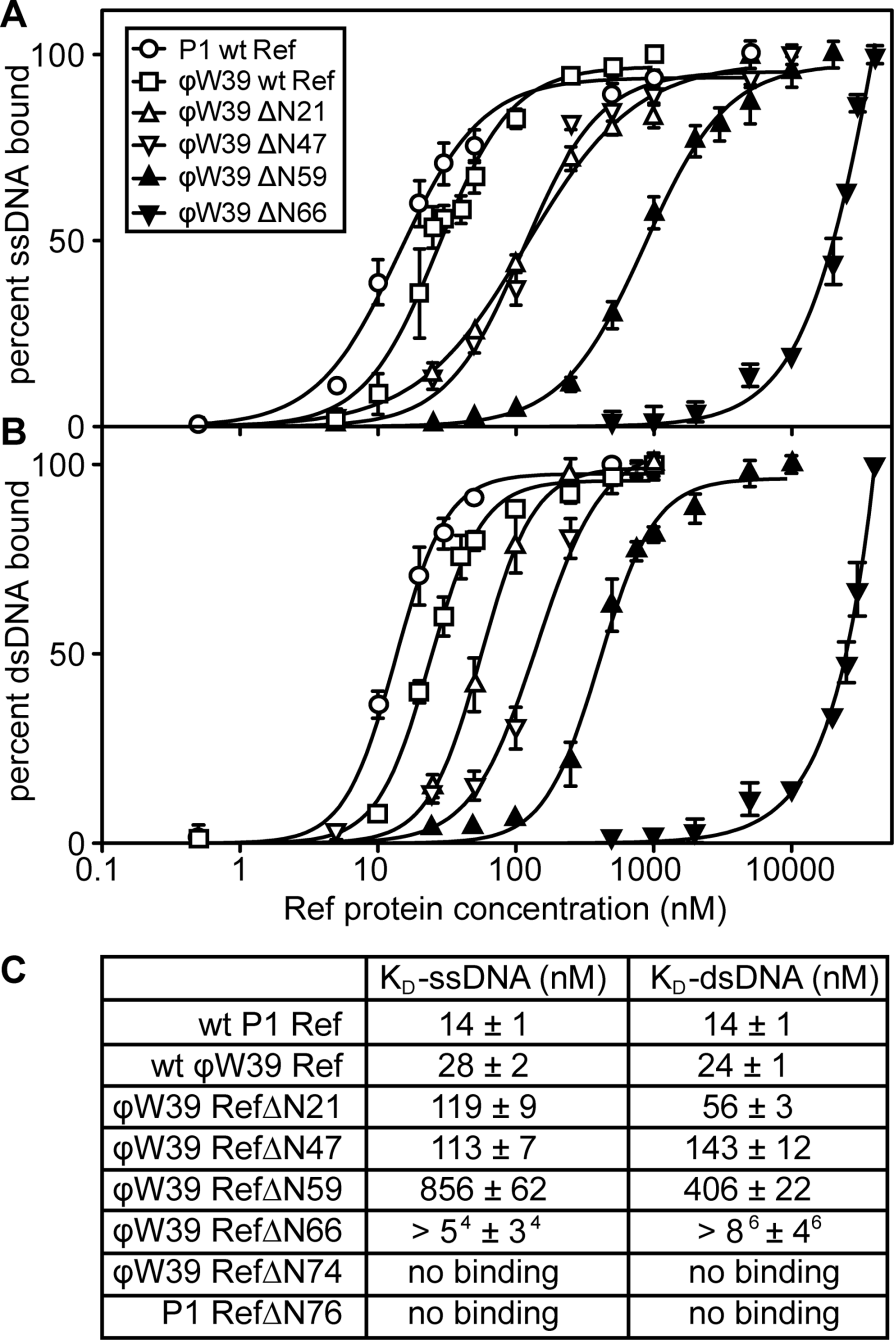
Ref truncations have distinct affinities for ssDNA and dsDNA in comparison to WT Ref. Equilibrium binding isotherms of WT Ref and truncations in presence of labeled ssDNA or dsDNA as monitored by fluorescence polarization. All data are the average of at least three experiments. Error bars are one standard deviation from the mean. (A) ϕW39 ΔN21 Ref and ϕW39 ΔN47 Ref show reduced affinity for ssDNA in comparison to WT Ref. (B) ϕW39 ΔN21 Ref and ϕW39 ΔN47 Ref also show a decreasing affinity for dsDNA as more of the N-terminus is removed in comparison to WT Ref. (C) Table of apparent *K*_d,app_ values for each Ref protein analyzed from at least three replicates. No DNA binding was observed with the ϕW39 ΔN74 Ref or P1 ΔN76 Ref.

When assayed with a labeled 71mer ssDNA (AJM25), the full-length P1 WT and ϕW39 WT Ref proteins exhibited similar *K*_d,app_ values (14 ± 1 nM and 28 ± 2 nM, respectively) (Figure 2A and C). It is possible that the small difference in DNA binding between the two proteins is due to the presence of a Glu instead of a Lys residue at position 13 in the ϕW39 WT Ref, which alters the first of the putative charge distribution motifs (Figure 1 and Supplementary Figure S1). However, upon removing one putative charge motif, the ϕW39 ΔN21 and ϕW39 ΔN47 Ref proteins exhibited a 6-fold reduction in binding (119 ± 9 nM and 113 ± 7 nM, respectively) (Figure 2A and C). Removal of the second charge motif in the ϕW39 ΔN59 resulted in a 40-fold reduction in binding affinity. The ϕW39ΔN66, which has a partial deletion of the third motif, exhibited further reduction in ssDNA binding, and DNA saturation was not attainable. Upon removal of the third charge motif in ϕW39 ΔN74 and P1 ΔN76 Ref proteins, DNA binding was no longer detectable (Figure 2C). Data for the P1ΔN76 Ref protein is consistent with Electrophoretic Mobility Shift Assay (EMSA) data from the earlier study (7). Our results suggest that each charge motif (or sequence features in or near these putative motifs) contributes to ssDNA binding. The results also suggest that the entire N-terminal 76 amino acid residue region contributes more or less additively to DNA binding.

The truncations were also assayed with a 71mer dsDNA substrate made by annealing the labeled 71mer oligonucleotide (AJM25) with the complementary 71mer oligonucleotide (AJG52). The P1 WT Ref and ϕW39 WT Ref proteins exhibited very similar *K*_d,app_ (14 ± 1 nM and 24 ± 1 nM respectively) (Figure 2B and C). Binding affinity for dsDNA was also reduced as the charge motifs were removed. However, ϕW39 ΔN21 Ref (56 ± 3 nM) and ϕW39 ΔN47 Ref (143 ± 12 nM) did not have similar *K*_d,app_ values, as seen with the ssDNA. This suggests a contribution to DNA binding from the region that separates the first and second of the putative charge distribution motifs. As the second motif and part of the third motif were removed in the ϕW39 ΔN59 and ϕW39 ΔN66, dsDNA binding was further reduced. As with the ssDNA, ϕW39 ΔN74 and P1 ΔN76 Ref had no observable dsDNA binding. Overall we provide evidence that the N-terminal region of Ref is necessary for DNA binding, and the putative charge motifs may contribute collectively (but probably not exclusively) to that function.

### Cys11 is necessary for disulfide formation and dimerization of Ref

**Figure 3.**
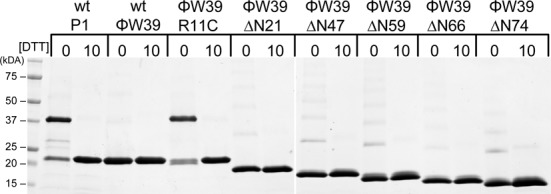
P1 Ref Cys11 is necessary for disulfide formation and dimerization of Ref. SDS-PAGE of dimerization assay in absence or presence of 10 mM DTT. WT P1 Ref and ϕW39 R11C Ref form stable dimers in non-reducing conditions, which are reduced in the presence of 10 mM DTT. WT ϕW39 Ref, ϕW39ΔN21 Ref, ϕW39ΔN47, ϕW39ΔN59, ϕW39ΔN66, ϕW39ΔN74 exhibit minimal formation of dimers and higher oligomeric complexes, and all are absent in the presence of 10 mM DTT.

We determined the oligomeric status of full-length Ref and all truncations. Preliminary ultracentrifugation data suggested P1 WT Ref to be a mixture of monomer and dimer, while the P1ΔN76 was only observed in monomeric form (unpublished data). We initially hypothesized that Ref functions as a dimer (it has only one active site but must cut each of two DNA strands in the targeted cleavage assay) and that the N-terminal region is important for Ref dimerization. Dimeric forms of P1 WT Ref were often observed on SDS-PAGE, but none of the truncated variants derived from ϕW39 Ref exhibited oligomeric properties. The non-specific cross-linking reagent, disuccinimidyl glutarate (DSG), was used to covalently trap the oligomeric states of Ref. The P1 WT Ref was again the only Ref that exhibited significant dimer formation (Supplementary Figure S2). Surprisingly, the ϕW39 WT Ref did not form a dimer even though it is indistinguishable from P1 WT Ref in DNA binding and other Ref nuclease assays. Upon amino acid sequence analysis of P1 WT and ϕW39 WT, we noted that P1 Ref contains Cys11 while ϕW39 Ref contains Arg11. In order to determine the role of Cys11, a ϕW39 R11C variant was constructed and purified. In the DSG cross-linking experiment, the ϕW39 R11C dimerized at levels comparable to WT P1 Ref (Supplementary Figure S2). This result suggested that a disulfide bond was responsible for oligomerization of Ref. The P1 WT Ref, ϕW39 WT Ref and all truncation variants were incubated in the absence or presence of 10 mM DTT and then run under non-reducing conditions on a SDS-PAGE. As shown in Figure 3, the P1 WT Ref and ϕW39 R11C exhibited a dimer under non-reducing conditions, while the ϕW39 WT Ref and truncation variants exhibited negligible amounts of oligomerization. DTT substantially reduced the dimerization of P1 WT Ref and ϕW39 R11C. The truncation variants’ monomeric state was verified by analytical size exclusion (data not shown). Although we previously hypothesized that the entire N-terminal domain was responsible for dimerization, these data provide evidence that Cys11 is the essential residue for dimerization of P1 Ref.

A recent model of Ref nuclease cleavage (8) hypothesized that Ref acts as a dimer due to the preliminary ultracentrifugation data from P1 WT Ref. We now conclude that the active form of Ref is monomeric. The targeted nuclease activity of P1 WT Ref and ϕW39 R11C Ref was not affected by the inclusion of 10 mM DTT (Supplementary Figure S3). Since the P1 WT Ref and ϕW39 WT Ref exhibit similar DNA cleavage kinetics in all of our assays, and the presence of DTT has no effect, it is likely that Ref is active as a monomer.

We probed for the presence of dimerization in two additional ways. First, we reasoned that dimers involving the catalytic domain might form during nuclease action in the presence of RecA protein. We thus carried out a targeted cleavage reaction in which the Ref ΔN74 variant (catalytic domain only) was added to the reaction mix 10 min after initiation with the WT Ref protein. The additional catalytic domains produced no notable effect on the progress of the reaction (Supplementary Figure S4). Second, we determined if dimers or other oligomeric states of Ref might form in the presence of ssDNA. ϕW39 WT Ref, ϕW39 Ref ΔN47 and ϕW39 Ref ΔN74 were incubated in the presence or absence of the 150 nt targeting oligonucleotide and plus and minus the cross-linking reagent DSG (Supplementary Figure S5). Cross-linking was quite modest in all cases, and the inclusion of ssDNA in the reaction produced no effect. Because the Ref protein is replete with primary amine residues the DSG should trap any higher oligomeric states. However, it is possible that higher order oligomeric states are not cross-linked in the presence of DSG.

### The N-terminus of Ref is necessary for ssDNA nuclease activity

**Figure 4.**
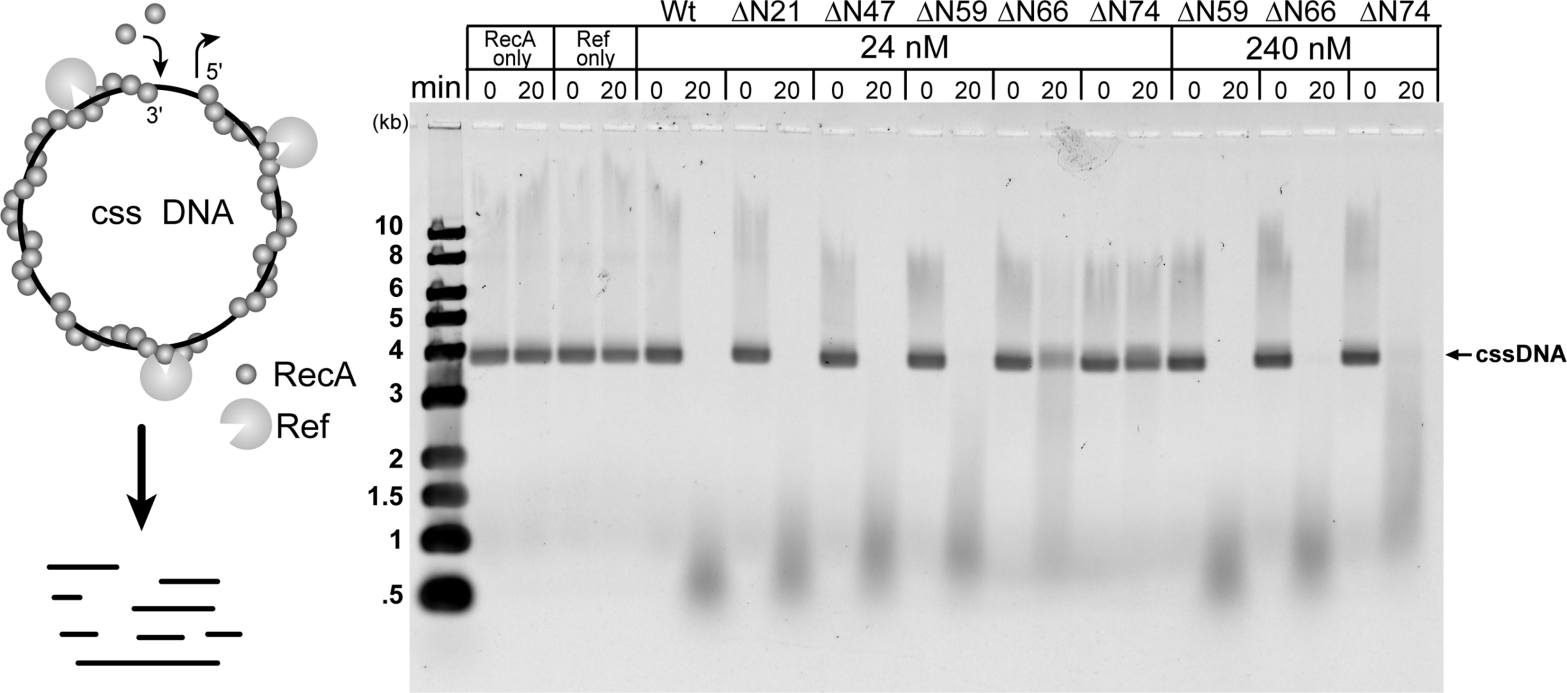
The amino acids between position 47 and 74 are important for Ref nuclease activity on circular ssDNA. (A) Schematic of Ref circular ssDNA nuclease assay, as described in Materials and Methods. (B) Agarose gel of reactions after 20-min incubation of circular ssDNA with all Ref variants. All circular ssDNA is degraded within 20 min with full-length WT ϕW39, ϕW39 ΔN21, ϕW39 ΔN47 and ϕW39 ΔN59 Ref. The ϕW39 ΔN66 and ϕW39 ΔN74 showed reduced activity at similar concentrations, but regained WT activity level with a 10-fold increase in protein concentration.

P1 Ref is a RecA-dependent nuclease on ssDNA (7). In the presence of M13mp18 circular ssDNA, RecA protein, ATP and Mg^2+^ there is evident degradation of the DNA within 20 min (Figure 4A), although the actual number of cleavage events per ssDNA is small (7). To test whether the truncations had any effect on nuclease activity, this assay was carried out using all truncation variants at the same concentration (24 nM). In the presence of RecA or Ref only there is no degradation of the circular ssDNA. After 20 min, the P1 WT, ϕW39 WT, ϕW39 ΔN21 and ϕW39 ΔN47 Ref all produced similar amounts of degradation in the presence of RecA (Figure 4B). However, there was a clear decrease in nuclease activity as the truncations progressed to ϕW39 ΔN59, ϕW39 ΔN66 and ϕW39 ΔN74 Ref (Figure 4B). The decrease activity gradient is consistent with the removal of the putative charge motifs. When concentrations of ϕW39 ΔN59, ϕW39 ΔN66, ϕW39 ΔN74 Ref were increased 10-fold, levels of degradation similar to those produced by WT Ref were observed (Figure 4B). This result suggests that the N-terminal domain does not alter the nuclease activity, but is necessary for Ref localization to the DNA and/or RecA nucleoprotein filament. The dramatic deficiency in the activity of ϕW39 ΔN66 and ϕW39 ΔN74 Ref coincides well with the deficiency in DNA binding affinity.

### Partial N-terminal Ref truncation enhances targeted double-strand cleavage

Using an assay established previously (8) we examined the efficiency of targeted dsDNA cleavage. Ronayne *et al.* (8) determined that the strand paired to the targeting oligonucleotide is cleaved first. This is followed by a mechanistically distinct cleavage of the displaced strand resulting in a linearization of the circular dsDNA (8). This work also showed that the targeting oligonucleotide was cut, but to a much lesser extent than the targeted circular dsDNA. The cleavage pattern and the degree to which the targeting oligonucleotide was cleaved was similar in the presence and absence of the circular dsDNA target (8). This suggests that the very limited cleavage of the oligonucleotide occurs independently of the targeting reaction (8).

**Figure 5.**
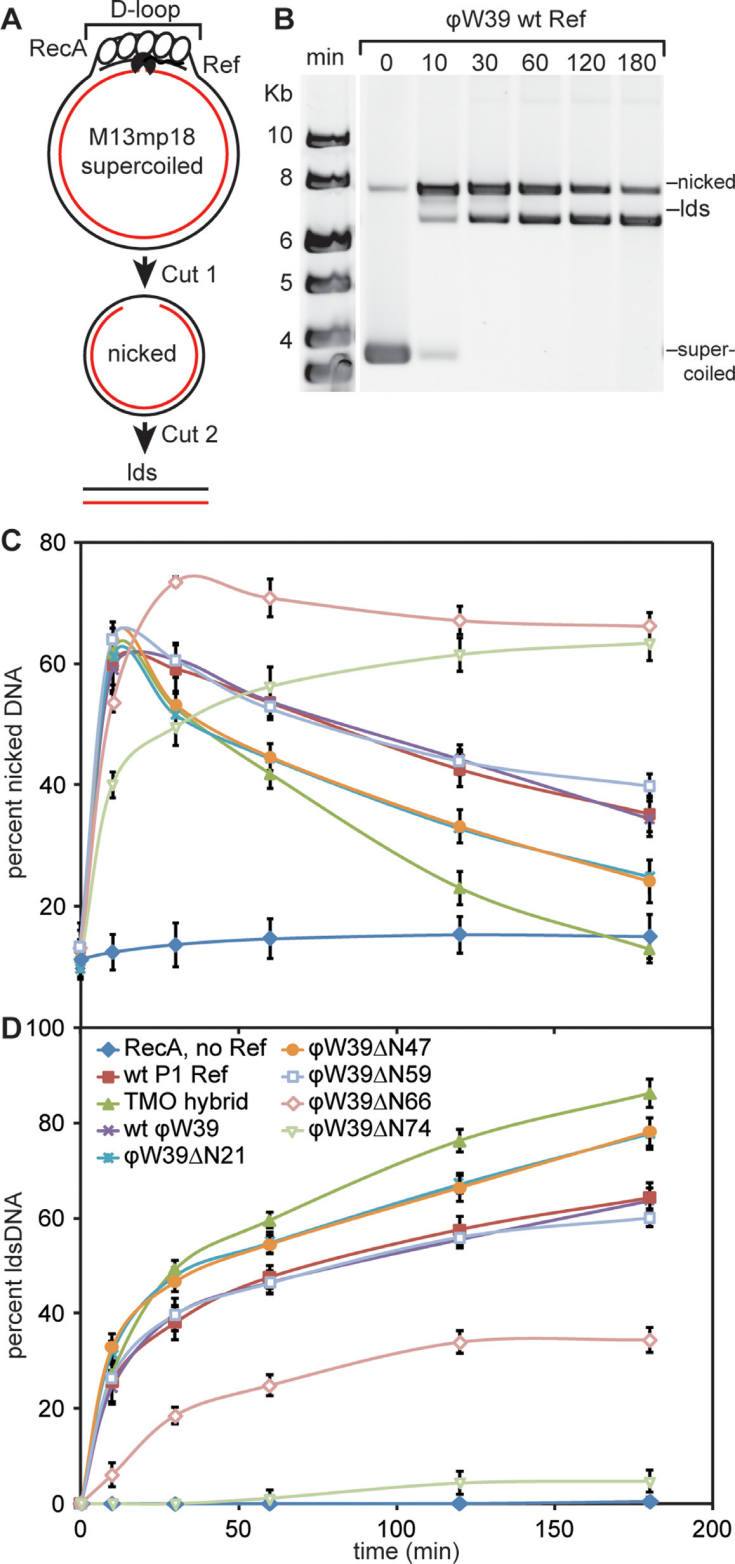
TMO hybrid Ref, ϕW39 ΔN21 Ref and ϕW39 ΔN47 Ref show increased efficiency in creating targeted DSBs. (A) Reaction schematic of nuclease site-specific targeting assay. (B) Agarose gel representing time points from the targeted nuclease assay with ϕW39 WT Ref. Supercoiled DNA is the starting material (bottom band), which is converted first to nicked DNA (top band) and subsequently to linear DNA (middle band). Reactions were carried out as described in Materials and Methods. (C) Graphical representation of nicked product formation over time, averaged over at least three independent experiments. At 10 min all Ref proteins exhibit similar amounts of nicking. At 30 min the TMO hybrid Ref, ϕW39 ΔN21 Ref and ϕW39 ΔN47 Ref show a greater decrease in nicked product, which corresponds to an increase in linear dsDNA formation due to the second cut on the displaced strand. (D) Graphical representation of linear dsDNA product formation over time, averaged over at least three independent experiments. Throughout the time course TMO hybrid Ref, ϕW39 ΔN21 Ref and ϕW39 ΔN47 Ref all exhibit enhanced production of linear dsDNA in comparison to WT Refs.

In the dsDNA targeting reaction, supercoiled M13mp18 circular dsDNA is incubated with a complementary 150-mer oligonucleotide and the RecA protein, which pairs the complementary oligonucleotide and duplex DNA (Figure 5A) to form a D-loop. Within the RecA-created D-loop, Ref cleaves the strand paired to the oligonucleotide rapidly, followed by a much slower cleavage in the displaced strand to create a 7.25-kb linear dsDNA (Figure 5A) (8). A representative agarose gel of the products formed in the presence of ϕW39 WT Ref is displayed in Figure 5B. The supercoiled circular dsDNA (Supplementary Figure S6), nicked circular dsDNA (Figure 5C) and linear dsDNA (Figure 5D) were measured as a percentage of the total DNA in each lane and followed over time.

The P1 WT, ϕW39 WT and ϕW39 ΔN59 proteins exhibited identical reaction kinetics with about 63% of supercoiled DNA being linearized (Figure 5D). Strikingly and reproducibly, the ϕW39 ΔN21, ϕW39 ΔN47 and TMO hybrid Ref increase linear dsDNA product formation by 15–20% (Figure 5D). At 30 min the ϕW39 ΔN21, ϕW39 ΔN47 and TMO hybrid Ref differed in kinetics compared to WT Refs by exhibiting a greater decrease in nicked DNA at 30 min (Figure 5C). This decrease in nicked DNA corresponded to the second cleavage event, producing linear dsDNA. The linear dsDNA timecourse corroborated this. At 30 min, we observed that the ϕW39 ΔN21, ϕW39 ΔN47 and TMO hybrid Ref produced about 10% more linear dsDNA than the WT Refs. This enhanced linear dsDNA product formation over WT proteins may be the result of a faster second cleavage event on the displaced strand.

In contrast, the ϕW39 ΔN66 and ϕW39 ΔN74 exhibited decreased targeted assay efficiency. This corresponded with a deficiency in DNA binding (Figure 2) and nuclease activity on circular ssDNA (Figure 4). The ϕW39 ΔN66 Ref had a 2-fold reduction in targeted nuclease activity, indicating that the removal of the 7 amino acids following the second charge motif is detrimental to the Ref activity. Upon removal of the remaining third putative charge motif, the ϕW39 ΔN74 Ref only generated 4% linear dsDNA product in 3 h. Both the ϕW39 ΔN66 and ϕW39 ΔN74 are capable of producing reduced but significant levels of nicked DNA, suggesting that the second cut needed to produce linear dsDNA is especially compromised.

## DISCUSSION

The present study provides an initial insight into the function of the intrinsically disordered N-terminal region of the Ref protein with two significant conclusions. First, the N-terminal region (i) is required for Ref binding to DNA, (ii) contains a Cys necessary for the observed dimerization unique to the P1 WT Ref and (iii) is necessary for efficient Ref-mediated DNA cleavage. Second, removal of part, but not all of the N-terminus increases the efficiency of targeted Ref cleavage at D-loops. The most efficient Ref variants examined in this study lacked either 21 or 47 N-terminal amino acid residues. In contrast, removal of additional amino acid residues led to WT levels of activity (ϕW39 ΔN59) or a dramatic decrease in Ref function (ϕW39 ΔN66 and ϕW39 ΔN74) as a targeting endonuclease.

Via its action at RecA-created D-loops, Ref cleavage is programmable and can be targeted to any DNA sequence. Exploiting this technology for genome editing and other potential biotechnology applications requires a detailed understanding of Ref's mechanism of action and structure-function relationships. Removal of between 21 and 47 amino acid residues from the N-terminus is a first optimization strategy for creating targeted DSBs *in vitro* with Ref. In the N-terminus, we have identified a possible motif consisting of a (consensus) ++..+..+–+ charge distribution pattern. This putative motif is found three times in the N-terminal region of Ref, although the repeats are imperfect (Figure 1A). The results indicate that removal of one repeat improves Ref function in the targeted cleavage assay, while removal of multiple motifs results in notable deficiency in function. We hypothesize that the removal of one repeat reduces the efficiency of direct binding of Ref to DNA, and thus reduces the DNA binding competition between Ref and RecA. High concentrations of Ref reduce targeted cleavage activity (E. Ronayne, personal communication), suggesting that competition between Ref and RecA for direct DNA binding may be detrimental. With more complete RecA filament formation on the targeting oligonucleotide, the nuclease function of Ref is more fully expressed. Amino acid sequence analysis of the Ref N-terminus did not reveal any known DNA binding domains such as helix-turn-helix, helix-hairpin-helix or NUMODs (20,21). Since the Ref protein does not display sequence-specific nuclease activity, the putative charge motifs we have identified may be involved in non-specific DNA interactions or are involved in making contacts within the RecA nucleoprotein filament groove or with DNA in the filament. We are uncertain if the charge motifs are necessary simply for direct electrostatic interactions with DNA/RecA or if, when bound to DNA and/or RecA filaments, they take on a structure necessary for Ref nuclease function.

Based on preliminary ultracentrifugation data on the P1 WT Ref (dimer-active) and P1ΔN76 (monomer-limited activity), we previously proposed that Ref functioned as a dimer. Although we now postulate that Ref is functionally monomeric, we cannot exclude the possibility that transient dimer formation occurs upon association with RecA or DNA. However, limited proteolysis in the absence or presence of DNA (data not shown) did not reveal a change in the protease sensitivity pattern. Cross-linking patterns with DSG are also not altered by the presence of DNA (Supplementary Figure S5). This suggests Ref does not change its oligomeric state in the presence of DNA.

**Figure 6.**
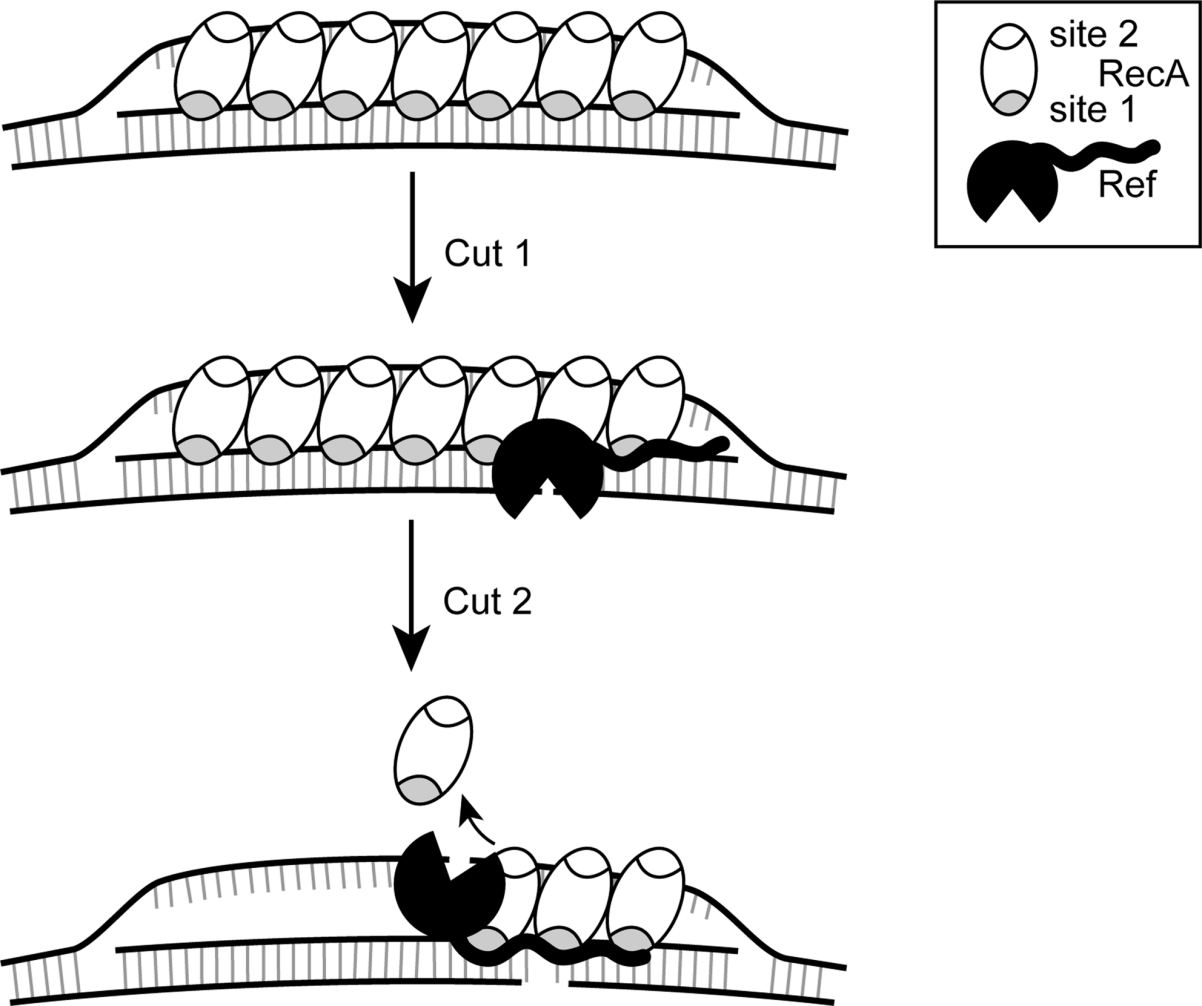
Revised model for the creation of targeted DSBs by Ref. RecA creates a D-loop by binding the targeting oligonucleotide in the primary binding site (gray), and after finding homology, binds the displaced strand in the secondary site (white). The N-terminal domain of Ref binds the paired strand or DNA within the RecA filament groove, positioning the Ref for the first cut. ATP hydrolysis-dependent disassembly of the RecA filament occurs in the 5′→3′ direction. Upon RecA disassembly, Ref can access the displaced strand and create cut 2. The N-terminal domain anchors the Ref protein and a rearrangement occurs to allow the C-terminal nuclease domain access to the displaced strand.

Combining the present results with previous work (8), we present an updated model for the production of targeted DSBs by Ref (Figure 6). The Ref protein cleaves the paired strand to create cut one and subsequently cleaves the displaced strand to produce a targeted DSB. As demonstrated previously (8), the first cleavage step does not require RecA-mediated ATP hydrolysis and can be promoted by the Ref active site mutant H153A. This suggests that the first cleavage of the paired DNA strand occurs within the RecA filament groove, and that residue H153 is not involved (8). Based on the present work, we suggest that the highly charged N-terminus of Ref acts as an anchor or tether, targeting the protein to the RecA protein groove. Access to the various DNA strands would then be modulated by the RecA protein structure within the D-loop. The slower, second cleavage event requires ATP hydrolysis and is not promoted by Ref H153A (8). This indicates that it may occur only during or after RecA filament disassembly (8). It also indicates that H153 is involved in this second cleavage event, suggesting that the active site is positioned somewhat differently than it is for the first cleavage event. Since this second DNA cleavage is as dependent on RecA as the first, we presume that Ref protein remains in contact with the RecA filament when carrying out this second cleavage. The Ref protein responsible could be the same one that promotes the first cleavage (after some type of rearrangement that may be reflected in the effects of the H153A mutation) or it could be a different Ref monomer that is also associated with the RecA filament. Once RecA filament disassembly occurs in proximity to the Ref protein, we hypothesize that the Ref monomer cuts the displaced strand (Figure 6). In Figure 6, we show one of these possibilities, involving a rearrangement of a single Ref monomer to allow separate consecutive cleavage events, although the involvement of two different Ref monomers is equally likely.

The two-domain structure of Ref, with an unstructured N-terminal DNA binding domain and globular nuclease domain, is similar to the HNH and GIY-YIG homing endonucleases I-HmuI and I-BmoI, respectively (22,23). We envision a similar cleavage mechanism to the GIY-YIG homing endonuclease, I-BmoI (22). I-BmoI functions as a monomer to create DSBs through two sequential strand cleavage steps (22). The DNA-binding domain could act as a molecular tether attached through a flexible linker to the GIY-YIG domain, permitting rotation of the nuclease domain to sequentially nick the DNA substrate on each strand, if indeed one monomer carries out both cleavage events. There have been significant advances in the resources available for genome editing, but there is room for expansion of this toolbox. The CRISPR/Cas9 genome editing system has seen abundant application in human cell lines, mice, zebrafish embryos, *Drosophila*, yeast and other model organisms (24–28). The genomic sequence of interest is targeted with a chimeric guide RNA (gRNA) (29,30) that directs genomic DNA cleavage by the endonuclease, Cas9 (29,30). The gRNA complementary sequence in the genome needs to be preceded by a NGG sequence, termed a protospacer adjacent motif (30). This modestly restricts sequences that can be targeted for editing. In addition, significant off-target cleavage has been observed in human cells, limiting some therapeutic and research applications (31). At present, the Ref system is limited by the need to introduce three components for cleavage (RecA, Ref and a targeting oligonucleotide). However, the Ref system has no target DNA sequence constraints. Due to the length of the oligonucleotide employed, non-specific cleavage of off-target sites can in principle be controlled.

## SUPPLEMENTARY DATA

Supplementary Data are available at NAR Online.

SUPPLEMENTARY DATA
