# Supplementary material for: Function of the N-terminal segment of the RecA-dependent nuclease Ref
Source: Nucleic Acids Res. 2015 Jan 23;43(3):1795–803. doi: 10.1093/nar/gku1330 (PMC4330346; doi:10.1093/nar/gku1330)
Supplement: SUPPLEMENTARY DATA [file supp_43_3_1795__index.html]

Function of the N-terminal segment of the RecA-dependent nuclease Ref — SUPPLEMENTARY DATA 

# Function of the N-terminal segment of the RecA-dependent nuclease Ref

## SUPPLEMENTARY DATA

**Files in this Data Supplement:**

- SUPPLEMENTARY DATA
