# Supplementary material for: Function of the N-terminal segment of the RecA-dependent nuclease Ref
Source: Nucleic Acids Res. 2015 Jan 23;43(3):1795–803. doi: 10.1093/nar/gku1330 (PMC4330346; doi:10.1093/nar/gku1330)
Supplement: SUPPLEMENTARY DATA [file supp_gku1330_nar-02403-h-2014-File008.pdf]

Supporting Information

SI Materials and Methods

DNA Substrates—Sequences of oligonucleotides used in this study are as follows: rlb1 150mer:

TTTTGGTTTTATCGTCGTCTGGTAAACGAGGGTTATGATAGTGTTGCTCTTACTATGCCTCGTAATTCCTT
TTGGCGTTATGTATCTGCATTAGTTGAATGTGGTATTCCTAAATCTCAACTGATGAATCTTTCTACCTGTA
ATAATGT

AJM25 71mer: 56FAM/CAGAAGTAAGTTGGCCGCAGTGTTATCACTCATGGTTATGGCAGCACTGCATA
ATTCTCTTACTGTCATGC

AJG52 71mer-
GCATGACAGTAAGAGAATTATGCAGTGCTGCCATAACCATGAGTGATAACACTGCGGCC
AACTTACTTCTG

Cloning Ref and Ref Variants- The WT *ref* gene from bacteriophage ϕW39 was not available, as stocks of this phage no longer exist. The gene was reconstructed to match the published sequence by mutagenesis of the P1 *ref* gene in pEAW584 (WT P1 *ref* in protein expression vector pET21A (Novagen)). The mutations were I8L, C11R, K13E, V34A, T46S, L47I, R86T, A93T, N107E, and A115S. Silent mutations to improve codon usage were also made and the resulting plasmid is pTMO9.

The ϕW39ΔN59 Ref – Plasmid pTMO9 was used as a PCR template with an upstream primer consisting of a NdeI site followed by bases 178-202 of ϕW39 *ref*, with a G to T change at base 192, and a T to C change at base 198 to make silent mutations for better codon use at codons 64 and 66. The ATG bases included in the NdeI site encode the first Met codon of the gene. The downstream primer consists of a BamHI site followed by bases 544-561 of the ϕW39 *ref* gene in 5′ to 3′ orientation. The PCR product was digested with NdeI and BamHI and inserted into pET21A digested with the same enzymes. The resulting plasmid, pEAW920 was directly sequenced to confirm the presence of ϕW39 ΔN59 Ref.

The ϕW39ΔN21, ΔN47, ΔN66, and ΔN74 Refs- Each plasmid was constructed in the same manner as pEAW920 except the upstream primers consisted of bases 64-101, 142-154, 199-222, 223-248, respectively of ϕW39 Ref. with several changes for improved codon usage at codons 49, 70, 75, 77, and 78 in one or more constructs. This generated plasmids pEAW886, pEAW865, pEAW919, and pEAW971, respectively.

The ϕW39 R11C Ref – Plasmid pTMO9 was used as the PCR template with an upstream primer consisting of a NdeI site followed by bases 4-45 of ϕW39 Ref, with the CGC coding for Arg at amino acid 11 changed to GCT to make a Cys substitution. The downstream primer consists of a BamHI site followed by bases 544-561 of the ϕW39 Ref gene in 5′ to 3′ orientation. The PCR product was digested with NdeI and BamHI and inserted into pET21A digested with the same enzymes. The resulting plasmid, pEAW933, was directly sequenced to confirm the presence of ϕW39 R11C.

TMO hybrid Ref vector is PTMO8, which encodes the P1 Ref protein in pET21A, with mutations at I8L, C11R, K13E, V34A, T46S, L47I, N107E, and A115S. It lacks the R86T and A93T mutations found in ϕW39 WT Ref.

Overexpression and Purification of Ref and Ref Variants:

The ϕW39 WT Ref and ϕW39 ΔN59 Ref were purified with procedures using similar growth, induction, cell harvesting, and early fractionation steps as P1 WT Ref. Both of these over-expression vectors were transformed into BL21(DE3) cells. Purification entailed polyethyleneimine precipitation, precipitation with $(NH_4)_2SO_4$, and chromatography successively using butyl-Sepharose (120 mL CV), Source 15 Q, Source 15S, and Sephacryl S-100 gel filtration columns. This was followed by another butyl-Sepharose (20 mL CV) chromatography step. Before cell lysis, the protease inhibitor Pefabloc SC (Sigma) was added to 0.1 mg/ml final concentration. Concentrations were determined using ProteinCalculator v3.4 calculated extinction coefficients of 2.788×10^4 M^{-1} cm^{-1} for ϕW39 WT Ref and 1.5220×10^4 M^{-1} cm^{-1} for ϕW39 ΔN59.

The ϕW39 ΔN21 Ref, ϕW39 ΔN47 Ref, ϕW39 ΔN66 Ref, ϕW39 ΔN74 Ref, and ϕW39 R11C Ref were purified similarly to the above proteins with one major modification: the overexpression vectors were transformed into BLR (DE3) cells. This prevents RecA contamination during the purification. Concentrations were determined using ProteinCalculator v3.4 calculated extinction coefficients of 2.219×10^4 M^{-1} cm^{-1} for ϕW39 ΔN21, 1.5220×10^4 M^{-1} cm^{-1} for ϕW39 ΔN47, ϕW39 ΔN66, ϕW39 ΔN74, and 2.788×10^4 M^{-1} cm^{-1} for ϕW39 R11C . All proteins were stringently tested for exonuclease and endonuclease contamination and all were free from detectable nuclease activity in the absence of RecA protein.

The TMO Hybrid Ref was purified with procedures using similar growth, induction, cell harvesting, and early fractionation steps as P1 WT Ref. Similarly, the over-expression vector was transformed into BL21(DE3) cells. Purification included polyethyleneimine precipitation, precipitation with $(NH_4)_2SO_4$, and chromatography successively using butyl-Sepharose (120 mL CV), hydroxyapatite, Source 15Q, and SP Fast Flow (GE17-5157-01) columns. These steps were followed by another butyl-Sepharose (20 mL CV) chromatography step. Significant degradation of the protein occurred during the hydroxyapatite step and successive chromatography steps were performed to purify the specific-sized degradation product lacking 47 N-terminal amino acid residues. Electrospray ionization and tandem mass spectrometry were used verify the amino acid position of degradation. Concentration was determined using ProteinCalculator v3.4 calculated extinction coefficient of 1.6500 x 10^4 M^{-1} cm^{-1}.

Circular ssDNA nuclease assay—Reactions are incubated with Buffer A (containing 25 mM Tris-acetate (pH 8.5), 3 mM potassium glutamate, 15 mM magnesium acetate, and 5%w/v glycerol), an ATP regeneration system (10 U/mL pyruvate kinase and 3.5 mM phosphoenolpyruvate), 4 μM nt M13mp18 cssDNA, and 2.4 μM RecA E38K at 37°C for 10 min. Reactions are incubated an additional 15 min with 3 mM ATP to allow an active RecA nucleoprotein filament to form prior to the addition of Ref at 24 nM (or as indicated). After 20 min, 20 μL of the reaction mixture are added to 10 μL of stop solution (9% Ficoll, 0.25% bromphenol blue, 0.25% xylene cyanol, and 4% SDS) then incubated another 30 min at 37 °C. Samples were analyzed by electrophoresis in 0.8% agarose with TAE buffer (40 mM Tris-Acetate, 1 mM EDTA), stained with SYBR gold, and visualized using the SYBR gold setting on a Typhoon FLA 9000 (GE Healthcare).

Nuclease site-specific targeting assay—The reactions were carried out at 37 °C in buffer A, (containing 25 mM Tris-acetate (pH 8.5), 1 mM DTT, 3 mM potassium glutamate, 15 mM magnesium acetate, and 5%w/v glycerol) and an ATP regeneration system (10 U/mL pyruvate kinase and 3.5 mM phosphoenolpyruvate. The above components were incubated for 10 minutes with a targeting oligonucleotide (4 μM total nucleotides, rlb1 150mer) and RecA E38K (1.33 μM). ATP (3 mM) was added and incubated an additional 20 minutes, followed by M13mp18 circular dsDNA (8 μMnt) and another 20 minute incubation. Before adding Ref, a zero time point was taken, then Ref (100 nM)

was added. The reactions were stopped at the noted time points by removing 20 μL from the reaction and adding it to 20 μL of stop solution (12 mM Tris acetate pH 7.5, 10.8% (w/v) Ficoll, 0.15% (w/v) each bromophenol blue and xylene cyanol, 8% SDS) and incubating at 37°C an additional 30 minutes. Samples were analyzed by electrophoresis on a 0.8% agarose gel with TAE buffer, stained with SYBR Gold stain, and imaged using the SYBR gold settings on a Typhoon FLA 9000 (GE Healthcare). Gel image was analyzed using ImageQuant TL software (GE Healthcare). Lanes were normalized for loading conditions by reporting individual band intensity as a percentage of the total band intensity in that lane.

Figure S1: Charge patterns in a consensus charge distribution motif. The pattern is imperfectly repeated. Three examples from two different Ref proteins are shown.

		Sequence position
**P1**	++..+..+–+	**12-21**
	++..+.+–+	**49-57**
	++...+.+.+	**64-73**
**ϕW39**	++–..+..+–+	**11-21**
	++..+.+–+	**49-57**
	++...+.+.+	**64-73**

Figure S2

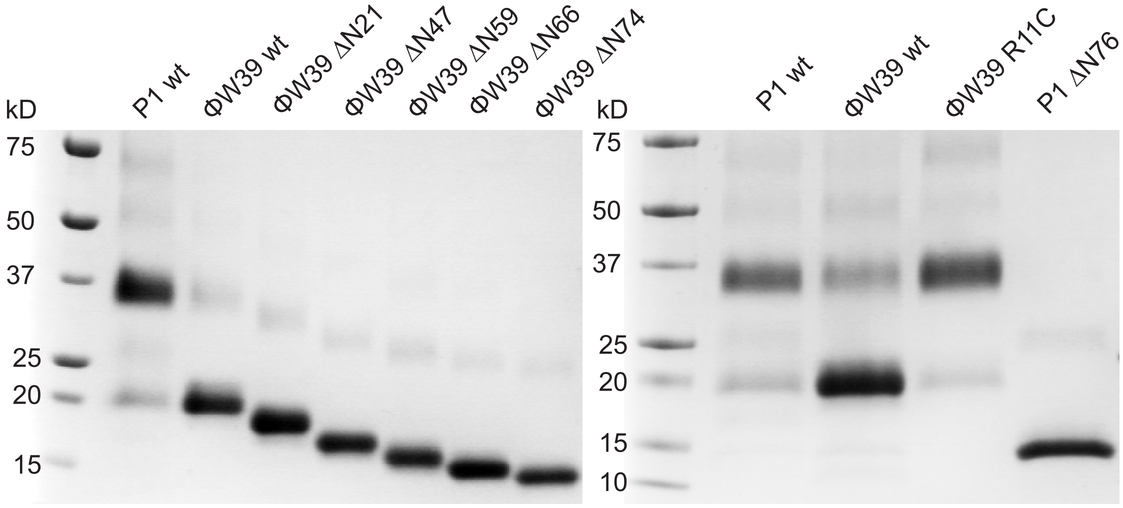

Figure S2: SDS-PAGE of Ref truncations and variants after treatment with disuccinimidyl glutarate (DSG), a non-specific primary amine crosslinker. The crosslinking covalently traps Ref in oligomeric states. P1 WT and ϕW39 R11C exhibit dimerization while WT ϕW39 Ref, ϕW39 ΔN21, ϕW39 ΔN47, ϕW39 ΔN59, ϕW39 ΔN66, ϕW39 ΔN74, and P1 ΔN76 show only background amounts of dimerization. Cys11 is responsible for dimerization of the P1 Ref protein.

Figure S3

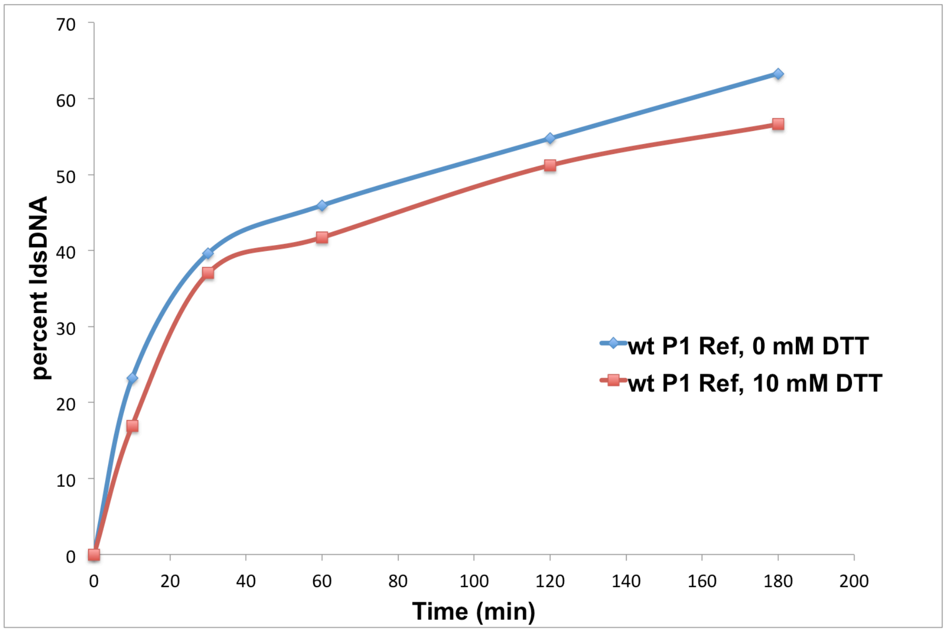

Figure S3: Graphical representation of ldsDNA formation over time from the nuclease site-specific targeting assay. Reactions were carried out as described in SI materials and methods, except 10 mM DTT was added immediately before Ref addition. P1 WT Ref was not affected by the inclusion of 10 mM DTT, indicating the Ref protein is functional as a monomer.

Figure S4

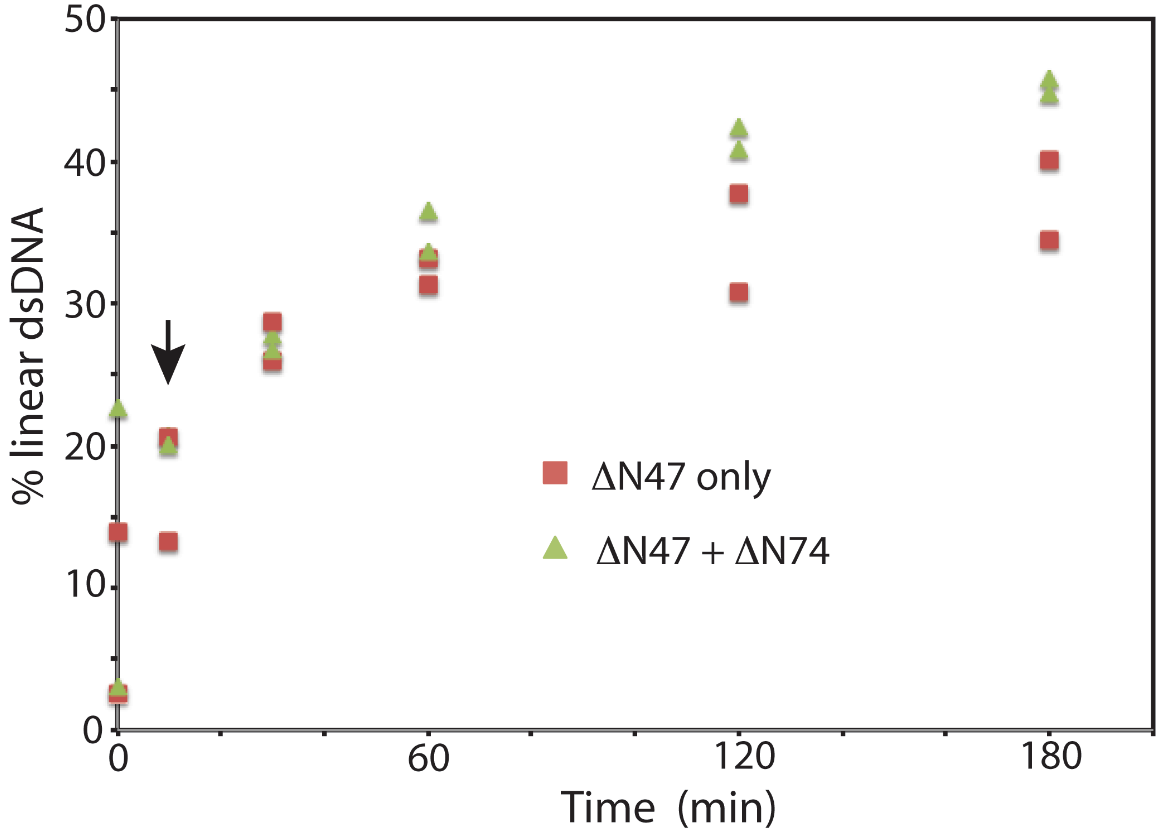

Figure S4. Effects of added Ref catalytic domains on an ongoing targeted DNA cleavage reaction. Targeted DNA cleavage was carried out as described in SI Materials and Methods (Nuclease site-specific targeting assay). The wild type RecA protein, which produces somewhat lower yields of Ref-mediated DNA cleavage events, was used in this experiment in place of RecA E38K. Reactions contained 100 nM ϕW39 Ref ΔN47. In two of the reactions (indicated in the figure), ϕW39 Ref ΔN74 was added at the ten-minute incubation period (arrow) at a concentration of 100nM. Reaction time points were taken at 0, 10, 30, 60, 120, and 180 minutes, and these were run on a 0.8% agarose gel. The gel was stained with SYBR-gold, imaged with Typhoon FLA9000, and the band percentages were quantified with ImageQuant (GE Healthcare) software.

Figure S5

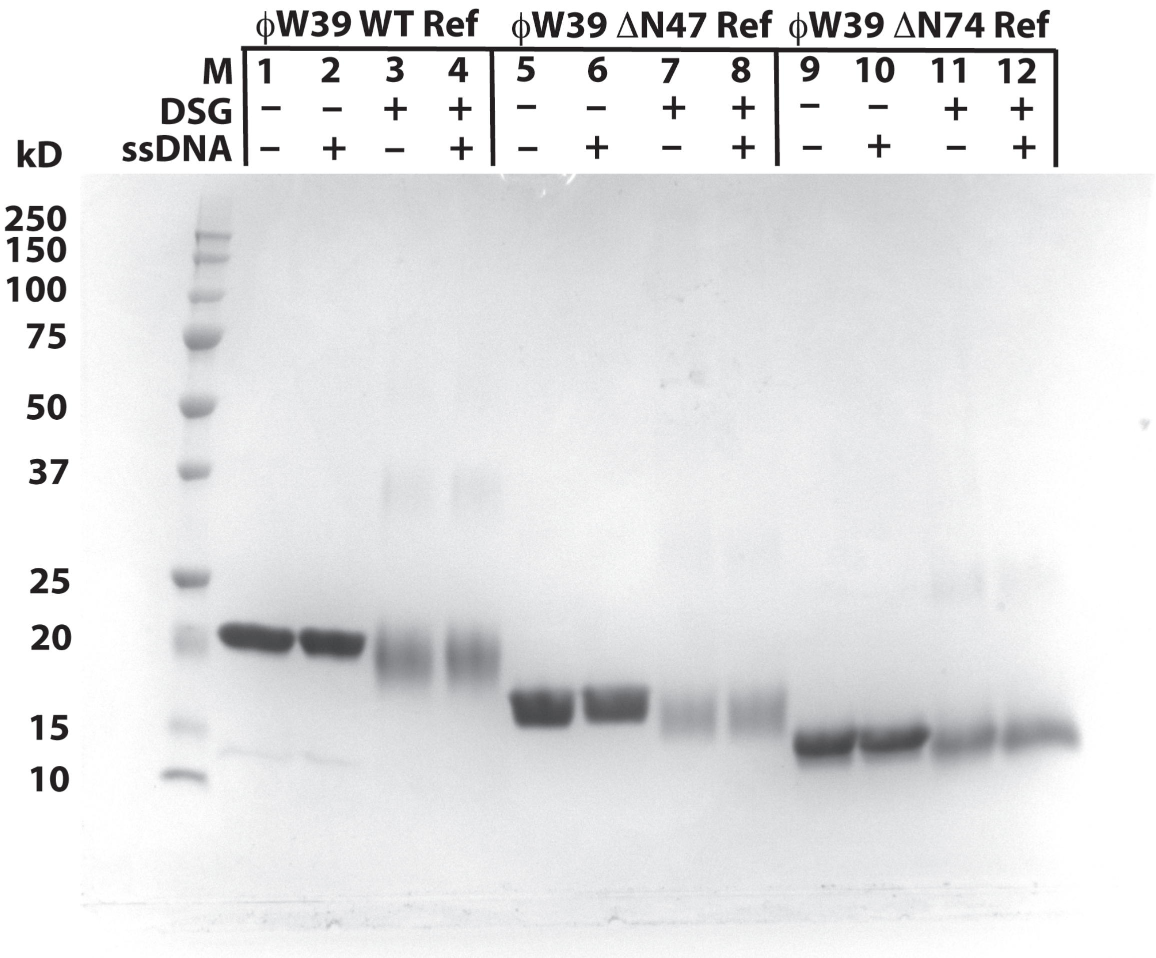

Figure S5 Ref Cross-linking Assay with and without DNA. Three Ref variants – ϕW39 Ref wildtype, ϕW39 Ref ΔN47, and ϕW39 Ref ΔN74– were each assayed at 15 μM concentration in 20 μL total volume reactions. This is 150X the standard Ref nuclease concentration used in the targeted DNA cleavage assays. All proteins were stored in phosphate buffer and the reaction buffer was also a phosphate buffer (50 mM $NaPO_4$, 75 mM NaCl, pH 7.5). These proteins were tested both with and without 0.67 μM (concentration given in total molecules) of the single-stranded 150-mer oligonucleotide used in the Ref cleavage assays. To make DSG solutions, 0.9 mg of DSG was dissolved in 50 μL DMSO. Reactions were also run with and without DSG crosslinking agent. The reactions were incubated for 10 minutes at 37 °C prior to the addition of 1 μL cross-linker or compensating DMSO as indicated. After the addition of DSG, the reactions were incubated for another 5 minutes before stopping the reaction with 3 μL of 1M Tris-HCl and 5 μL of 5x cracking buffer (400 mM Tris 80% cation or pH 6.8; 10% sodium dodecyl sulfate; 50% glycerol; 500 mM dithiothreitol; 1% bromophenol blue; 500 mM β-mercaptoethanol). The reactions were heated at 90 °C for 5 minutes before loading 12 μL of each reaction on a 12% SDS-Page gel. The gel was run for 1 hour at 120 mV.

Figure S6

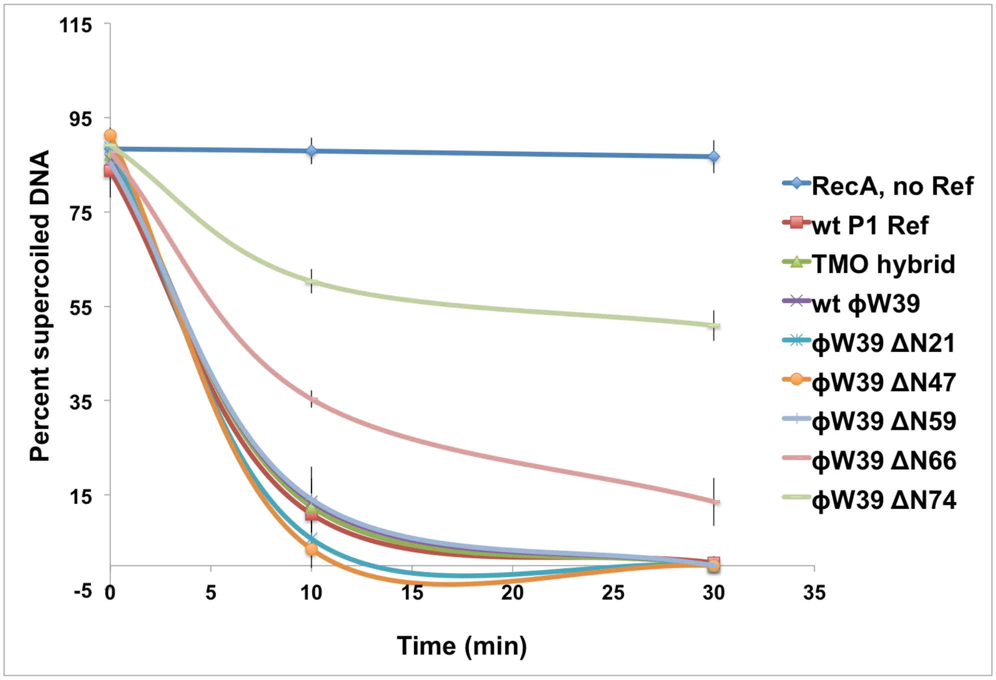

Figure S6: Graphical representation of supercoiled DNA disappearance over time from the nuclease site-specific targeting assay. Reactions were carried out as described in SI materials and methods. P1 WT Ref, WT ϕW39 Ref, TMO hybrid, ϕW39 ΔN21, ϕW39 ΔN47, and ϕW39 ΔN59 show similar kinetics in the first 30 minutes. The ϕW39 ΔN66 and ϕW39 ΔN74 are much slower in comparison. Overall this suggests the first cut to produce nicked DNA, is not different between the Ref proteins tested.
